# Retrospective comparison of three commercial artificial intelligence algorithms for detection of intracranial hemorrhage (ICH) in the emergency radiology department

**DOI:** 10.1177/02841851261484026

**Published:** 2026-09-15

**Authors:** Kristoffer Järlevi, Daniel Lindbom, Andreas Lindholm, Gustav Alvfeldt, Chikako Suzuki, Michael L Wilczek

**Affiliations:** 1 Department of Diagnostic Radiology, 59570 Södersjukhuset, Stockholm, Sweden; 2 Department of Clinical Science, Intervention and Technology, 27106 Karolinska Institutet, Stockholm, Sweden; 3 27106 Department of Molecular Medicine and Surgery, Karolinska Institutet, Stockholm, Sweden

**Keywords:** intracranial hemorrhage, artificial intelligence, computed tomography, clinical validation

## Abstract

**Background:**

Several commercial artificial intelligence (Al) algorithms are available for detecting intracranial hemorrhage (ICH), but independent clinical validation remains limited.

**Purpose:**

To compare three commercially available Al algorithms for ICH detection on non- contrast head computed tomography (NCHCT).

**Material and Methods:**

In this retrospective study, 4027 consecutive NCHCT examinations from a large emergency hospital in southwest Sweden were analyzed. Three Al algorithms were applied, with one vendor disclosing participation. Reports from two radiologists and all Al outputs were evaluated. Human-AI performance was assessed using an idealized logical OR model, assuming radiologists perfectly dismissed all false-positive Al flags to calculate system specificity. All positive or discrepant cases underwent expert manual review using two-tier consensus adjudication as the reference standard.

**Results:**

Of 3902 evaluable examinations, 3517 were consistently negative by all readers. The remaining 385 cases underwent manual review, confirming ICH in 176 cases (4.5% prevalence) and excluding it in 209. Eight ICH cases missed by both radiologists were detected by at least one Al system. Aidoc performed best, with 90.3% sensitivity and 99.0% specificity. A simulated mathematical combination of Aidoc with a human reader increased sensitivity to 96.0% while maintaining 99.4% specificity (*P* < 0.001), comparable to two radiologists.

**Conclusion:**

Al performance varied substantially, with only one system demonstrating clinically relevant accuracy. Combining Al with human interpretation improved ICH detection and shows promise for future clinical implementation.

## Introduction

Intracranial hemorrhage (ICH) is among the most extensively investigated applications of clinical artificial intelligence (AI). A recent systematic review including both commercial and non-commercial algorithms reported that AI-based neuroimaging tools can achieve high sensitivity and specificity.^1^ In contrast, a comprehensive evaluation of 100 commercially available radiology AI products found that only 36 had peer-reviewed evidence published regarding their clinical efficacy, with the majority of those studies demonstrating lower levels of support.^2^ Furthermore, a recent meta-analysis highlighted significant methodological limitations in AI research for first-line neuroimaging,^3^ showing that most studies on abnormality detection failed to meet fundamental scientific standards or employ robust validation strategies, thereby restricting their clinical relevance. Comparative studies using identical datasets remain scarce.^4–8^ It is also important to note that CE marking and FDA approval do not equate to proven clinical applicability.^9^

The use of computed tomography (CT) has risen markedly over the past two decades, with a 130% overall increase reported in Sweden between 2005 and 2018,^10^ and a 500% rise in head CT examinations observed between 2006 and 2020 at a large hospital in the Netherlands.^11^ This escalating demand, coupled with a shortage of experienced radiologists, places considerable strain on healthcare systems and heightens the risk of diagnostic errors. The integration of AI into clinical workflows has therefore been proposed as a potential strategy to address these challenges.^12^

The aim of the present study was to assess the performance of three commercially available AI algorithms for the detection of intracranial hemorrhage.

## Material and Methods

This retrospective, single-center cohort study was conducted at the Department of Radiology of a large emergency care hospital in Stockholm, Sweden, providing comprehensive services across all medical specialties. The hospital has a neurological but not neurosurgical department. The study protocol was reviewed and approved by the Swedish Ethical Review Authority (Dnr 2022-067730-01), which waived informed consent. All data were de-identified.

All patients who underwent acute non-contrast head CT (NCHCT) between 1 August and 31 December 2022 were eligible for inclusion. A total of 4027 consecutive NCHCT examinations were initially identified as potentially eligible. To ensure the analysis reflected first-line imaging, only the initial NCHCT scan was included when multiple scans were identified for a single patient. Patients who had undergone a head CT within 2 months before data collection were also excluded. Neoplasms of any etiology were manually excluded from the final cohort to ensure data homogeneity. Figure 1(a) depicts a flow diagram following EQUATOR/STROBE guidelines outlining the number of scans evaluated for eligibility, the exclusions with corresponding reasons, and the final analytic groups.

**Figure 1.**
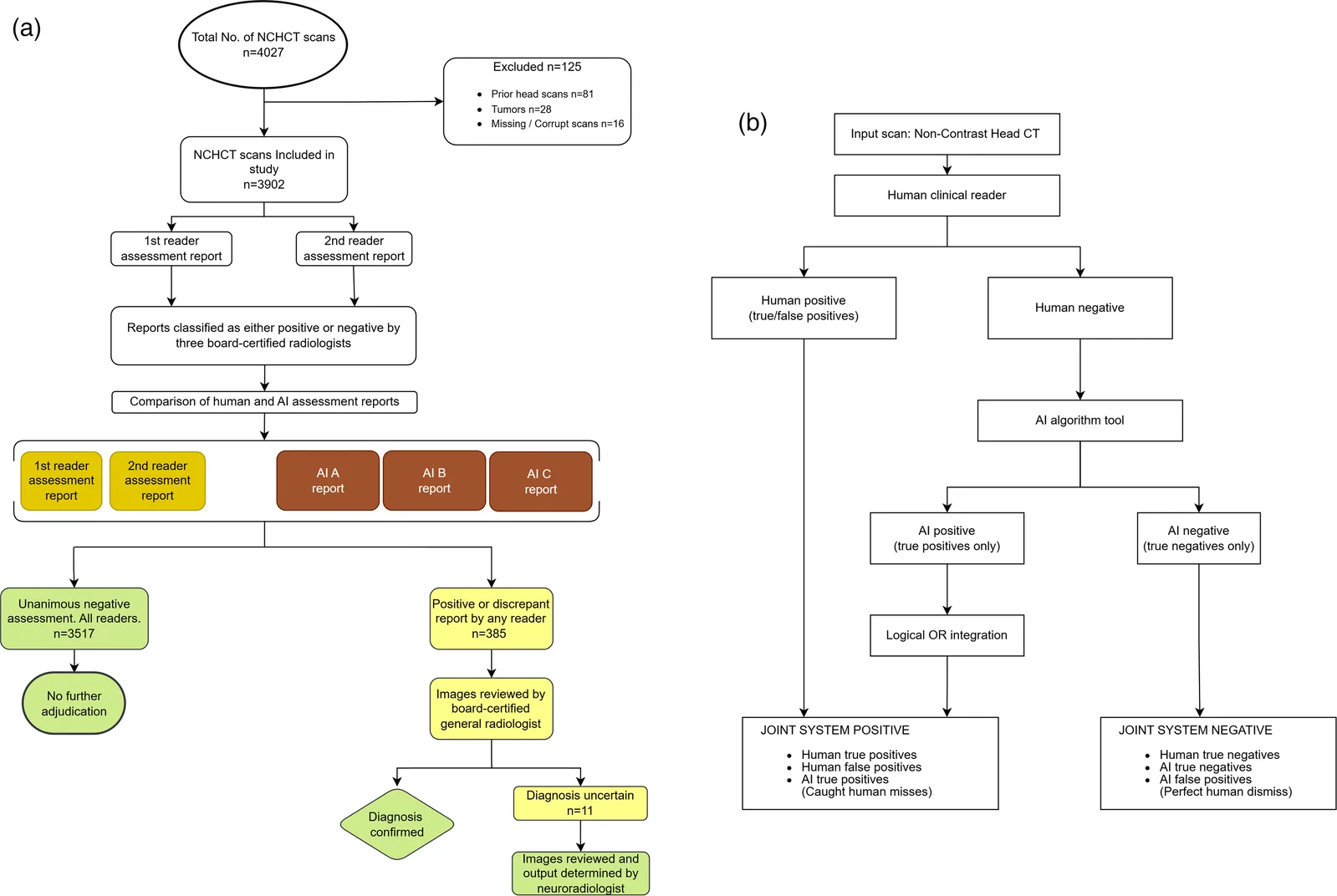
(a) Study inclusion flowchart and reference standard adjudication. Flow chart tracking the inclusion and exclusion criteria of the 4027 initial NCHCT scans, alongside the downstream expert adjudication pathways for the 385 positive or discrepant cases. (b) Idealized human-AI joint decision pathway. Schematic block diagram modeling the operational logic of the combined human-AI system. The model utilizes a logical OR configuration where final positive determinations are triggered by human reads or standalone true AI marks, while automated false-positive flags are assumed to be safely dismissed upon subsequent human review. AI: artificial intelligence; NCHCT: non-contrast head computed tomography.

### CT protocol

All patients underwent NCHCT as part of the emergency evaluation for possible acute brain injury. Examinations were performed on multidetector CT (MDCT) systems without dual-energy, using a 120-kV brain protocol on Somatom Drive, Somatom Definition Edge, and Somatom Definition Flash (Siemens Healthineers, Erlangen, Germany).

Acquisition parameters were as follows: detector configuration = 40 × 0.60 mm, tube voltage = 120 kV, 350 quality reference mAs, Care kV set to semi mode, pitch = 0.5, and rotation time = 0.5 s. Patients were positioned supine, head first, with the head secured in a dedicated head holder. Scan acquisition was aligned parallel to the infraorbitomeatal line, with coverage extending from the C1 level to the vertex.

Images were reconstructed with a slice thickness of 0.75 mm and an increment of 0.5 mm using a standard convolution kernel. Multiplanar reconstructions were generated in axial planes (5 mm and 2.5 mm), as well as coronal and sagittal planes (3 mm and 1.5 mm), in all cases. All imaging data were archived in the institutional picture archiving and communication system (PACS; Sectra, Linköping, Sweden) and subsequently distributed to the three AI vendors for analysis.

### AI-algorithms

Three commercially available CE-marked and FDA-cleared AI algorithms (designated as AI-A, AI-B, and AI-C (Aidoc Medical Ltd., Tel Aviv, Israel) for ICH detection were evaluated using default, vendor-specified operating thresholds. All three algorithms are actively utilized in standard clinical practice globally, but none of the three platforms were part of our local institution's standard clinical imaging infrastructure during the study period. They were evaluated purely within the scope of this retrospective research framework. Aidoc Medical Ltd. consented to disclosure, whereas the other two requested anonymity due to commercial confidentiality. The dataset was provided to each vendor in Digital Imaging and Communications in Medicine (DICOM) format, and the output from each AI was collected. In addition, vendors supplied key images illustrating AI-detected ICH, displayed using algorithm-specific visualization methods such as heatmaps or annotated regions of interest (ROIs).

### AI training and networking

All three algorithms were taught using supervised deep learning and retrospective datasets of de-identified CT examinations annotated by board-certified radiologists. Convolutional neural network architectures (CNNs) designed for volumetric medical imaging were employed. Training, validation, and testing were performed on independent datasets from multiple institutions and scanner vendors. AI performance was evaluated using separate retrospective and clinical validation datasets.

### Scan interpretation and adjudicated reference standard

The initial NCHCT interpretation was performed by the on-call radiologist, hereafter referred to as “reader 1” (typically a radiology resident; n = 49). Per our institutional clinical workflow, all examinations underwent subsequent double reading by a board-certified radiologist within a few days, hereafter referred to as “reader 2” (n = 30).

For study purposes, three board-certified general radiologists (KJ, MW, and DL) retrospectively evaluated and categorized all text-based clinical reports, split evenly among them. The three radiologists did not review the raw CT scan images during this phase. Instead, they evaluated the written text of the reports to establish positive or negative status. Because the text reports were divided evenly and reviewed independently without overlap; multi-reader kappa values for the three report-categorizing radiologists were not calculated.

The human findings were compared with the outputs of each individual AI algorithm. Examinations unanimously reported as negative—defined as no ICH detected by both clinical radiologists and all three Al algorithms—were classified as true negative without further adjudication. When multiple head scans for a single patient were found, only the first scan was included in the analysis. In cases presenting with multiple ICHs, the detection of at least one hemorrhage by a reader or algorithm was sufficient for classification as a positive case.

To establish the final reference standard, any examination reported as positive or inconclusive by any of the clinical readers or flagged as positive by any of the three Al algorithms (totaling 385 cases) underwent secondary manual image review. These discrepant or positive cases were resolved via a two-tier consensus adjudication process. The images were manually reviewed by a general radiologist with 7 years of experience (KJ); any persistent diagnostic uncertainty was definitively resolved by a subspecialized neuroradiologist with 3 years of clinical experience. Figure 1(a) details the patient cohort selection and manual adjudication tracking.

### Human-AI combination

The human-AI combination was evaluated using an idealized model, where a case was classified as positive if an ICH was identified by the human reader (true positives and false positives) or (logical OR) correctly identified by the AI (only true positives). All other cases were classified as negative including false positive cases identified by AI. Figure 1(b) illustrates the idealized workflow and its joint decision-making logic.

### Statistics

For pairwise comparisons of sensitivity and specificity between different methods of assessment, one-sided exact binomial tests were used. Positive predictive value (PPV), negative predictive value (NPV), and diagnostic odds ratio (DOR) were compared using relative predictive values developed by Moskowitz and Pepe.^13^ To assess the degree of agreement between the different interpreters beyond what would be expected by chance, Cohen's kappa coefficient was calculated as (κ = [P_0_ - P_e_]/[1 - P_e_]), where P_0_ is observed agreement and P_e_ is expected agreement by chance. Kappa values were interpreted as poor (0–0.20), fair (0.21–0.40), moderate (0.41–0.60), substantial (0.61–0.80), or almost perfect (0.81–1.00).^14^ Agreement patterns were examined using 2 × 2 contingency tables. Unless stated otherwise, continuous demographic and clinical data are presented as mean ± SD. All calculations were made in R version 4.3.2 (R Foundation for Statistical Computing, Vienna, Austria). *P* values <0.05 were considered statistically significant.

## Results

### Patient characteristics

A total of 3902 patients (1887 men (49%), 1991 women (51%); mean age = 65 ± 20 years; age range = 18–102 years) were included in the final cohort. Age distributions were comparable between male (64 ± 20 years) and female (66 ± 21 years) subgroups. Sex was unknown for 24 patients in the total cohort, all of whom had negative imaging findings.

Acute pathologies were identified on 176 scans, yielding an overall positive scan rate of 4.5%. Of the patients presenting with positive findings, a higher proportion were male (n = 106, 60%) compared to female (n = 70, 40%). Conversely, negative scans accounted for 95.5% (n = 3726) of the total cohort, consisting of 1781 (48%) men and 1921 (52%) women.

Among the 176 positive scans, a total of 256 individual radiological findings were recorded, indicating that a subset of patients presented with concurrent, multi-subtype intracranial pathologies. Subarachnoid hemorrhage was the most frequently observed subtype, accounting for 37.5% (n = 96) of total findings. This was followed closely by subdural hematomas at 34.4% (n = 88). Intraparenchymal hemorrhages and contusions comprised 16.8% (n = 43) and 10.9% (n = 28) of the findings, respectively. Epidural hematomas were rare (n = 1, 0.4%), and no cases of primary isolated intraventricular hemorrhage (n = 0) were documented. We acknowledge that trace intraventricular extension occurred secondarily within our massive SAH and intraparenchymal cohorts. Sex-specific breakdowns were not evaluated for individual pathology subtypes (Table 1).

**Table 1.** Basic demographic and clinical characteristics of the patient cohort (n = 3902).

	Total	Men	Women
Patients^a^	3902	1887 (49)	1991 (51)
Age (years)	65 ± 20 (18–102)	64 ± 20 (18–101)	66 ± 21 (18–102)
Scan image findings^b^			
Negative	3726 (95.5)	1781 (48)	1921 (52)
Positive (total patients)	176 (4.5)	106 (60)	70 (40)
Positive scan image findings subtypes	n = 256 total findings		
Subarachnoid hemorrhage	96 (37.5)	–	–
Subdural hematoma	88 (34.4)	–	–
Intraparenchymal hemorrhage	43 (16.8)	–	–
Contusion	28 (10.9)	–	–
Epidural hematoma	1 (0.4)	–	–
Intraventricular hemorrhage^c^	0 (0.0)	–	–

Values are given as n (%) or mean ± SD (range). Gender-specific breakdowns were not recorded for individual pathology subtypes.

a Sex was unknown for 24 patients, all of whom had negative imaging findings.

b Individual positive scan findings (n = 256) outnumber the total number of patients with positive scans (n = 176). This discrepancy is due to the presence of concurrent, multi-subtype pathologies within single patient scans (findings are not mutually exclusive).

c No cases of primary isolated intraventricular hemorrhage were documented. Trace intraventricular extension occurred secondarily within our massive SAH and intraparenchymal cohorts.

### Reader performance

A total of 3902 examinations were evaluated. Of these, 3517 were consistently interpreted as negative by both radiologists and all three AI algorithms. The remaining 385 examinations underwent adjudication (Figure 1), confirming 176 ICH cases and 209 negatives. AI algorithms flagged 24 ICH cases missed by reader 1, of which 16 were subsequently identified by reader 2. Eight cases were missed by both radiologists but detected by at least one AI algorithm. Table 2 presents the number of reader 1-missed and reader 2-missed ICH cases identified by each AI algorithm. Figures 2 and 3 illustrate examples of a reader 1-missed ICH that was subsequently detected by an AI algorithm.

**Figure 2.**
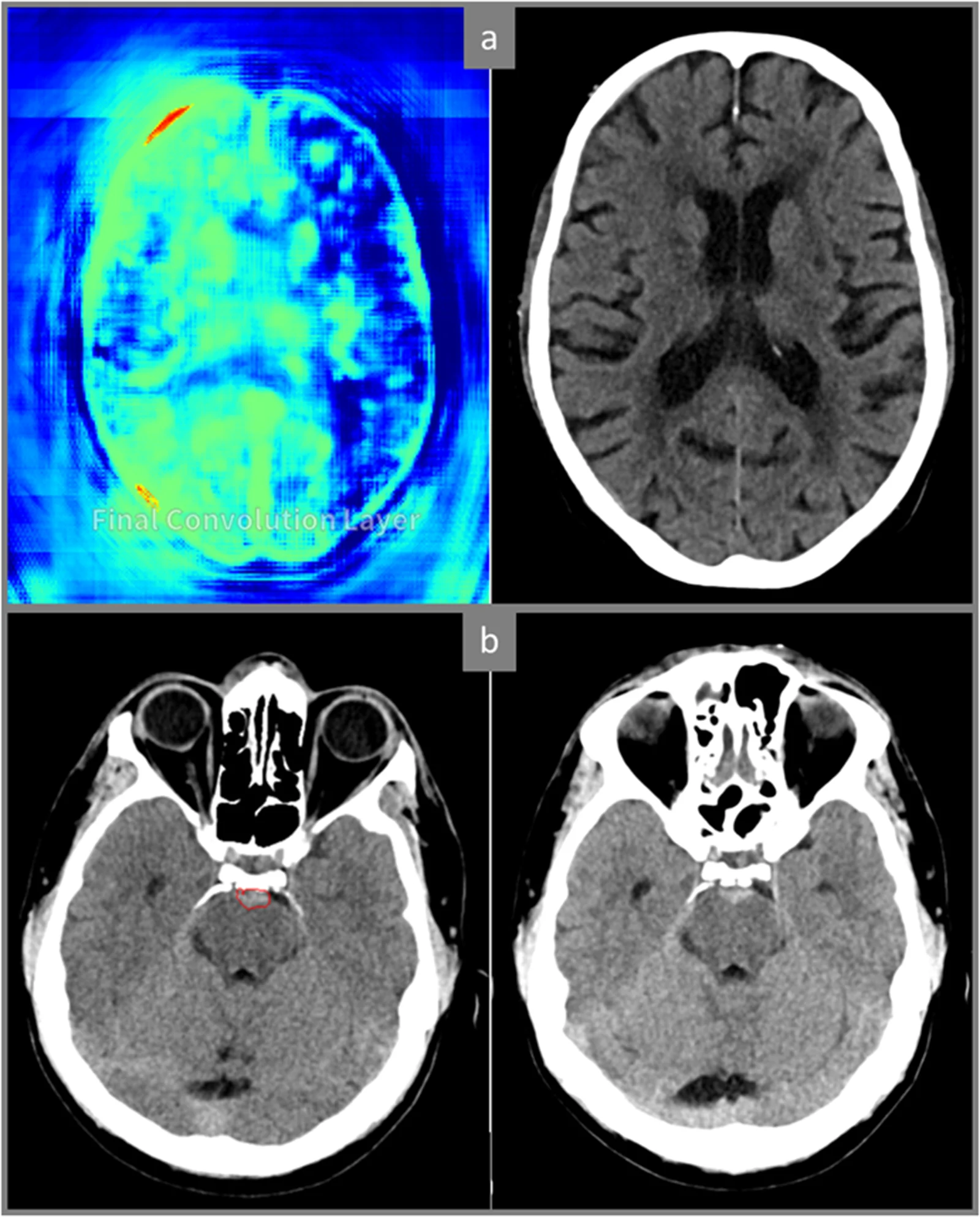
Examples of AI positives missed by reader 1. (a), (b) Images to the left show different AI overlays. (a) (b) Images to the right (a, b) are the corresponding originals to the AI overlays.

**Figure 3.**
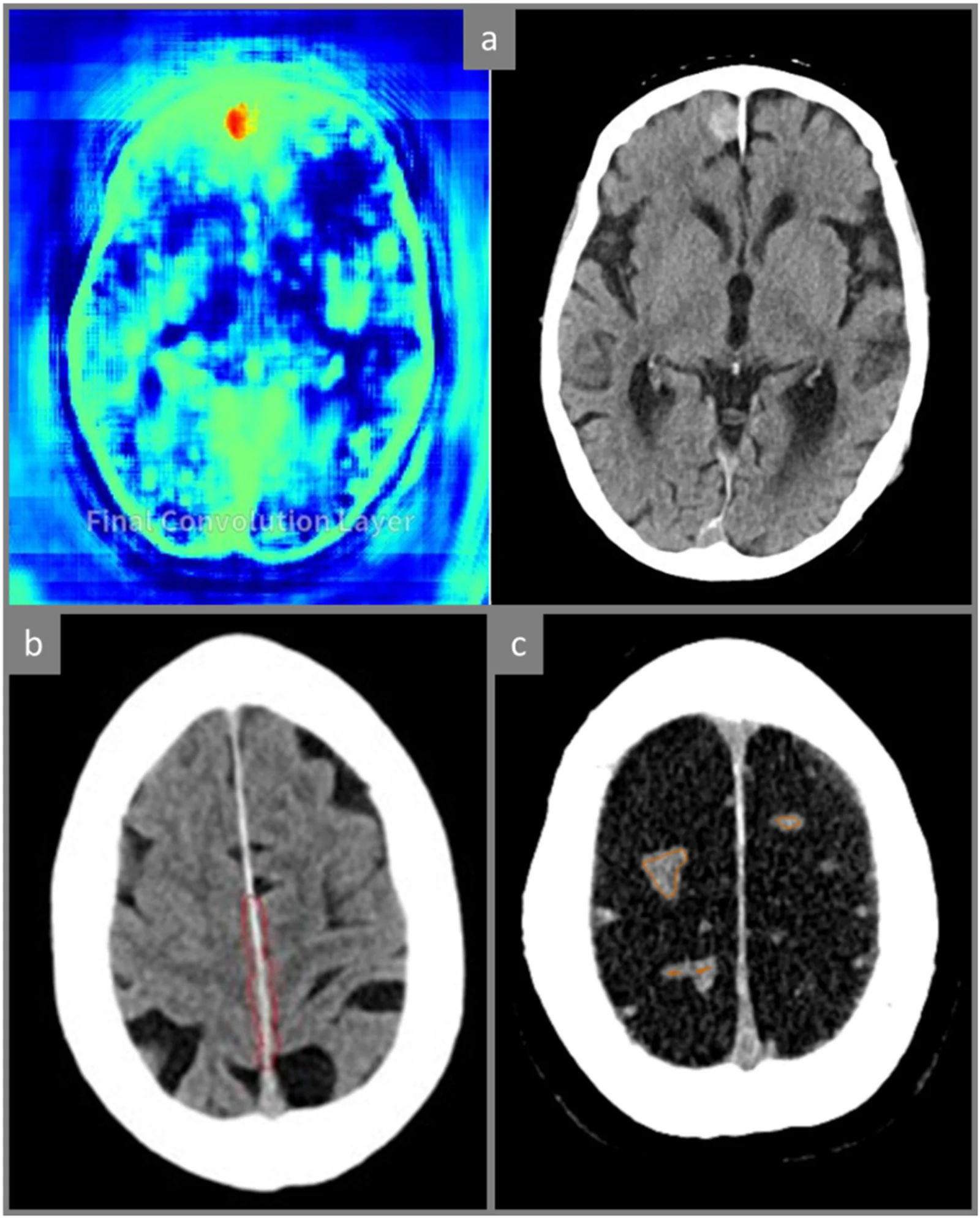
Examples of AI false positives, often easily dismissible by human readers. (a) Top: the same examination with AI overlay and corresponding original. (b), (c) Bottom: different examinations with AI overlays.

**Table 2.** The number of ICHs missed by readers 1 and 2, and the numbers of ICHs detected by each algorithm.

	ICH missed by human reader	ICH detected by AI-A		ICH detected by AI-B		ICH detected by AI-C	
	n	n	(%)	n	(%)	n	(%)
Reader 1	24	3	(13)	3	(13)	17	(71)
Reader 2	8	2	(25)	3	(38)	6	(75)

Counts represent unique patient examinations. If an algorithm detected multiple distinct hemorrhages within a single patient missed by a reader, it is counted as one unique patient case to maintain alignment with Table 3.

AI: artificial intelligence; ICH: intracranial hemorrhage.

### AI performance and comparative results

AI-C achieved the highest sensitivity of 90.3% and a specificity of 99.0%, whereas AI-A showed the lowest sensitivity (61.4%) and specificity (97.2%). Reader 1 had a sensitivity of 86.4% and reader 2 had a sensitivity of 95.5%. The corresponding specificity was 99.4% and 99.5%. Although AI-C had sensitivity comparable to reader 1, its PPV was lower (80.3% vs. 87.9%) due to almost twice as many false-positive results (Table 3), often representing clear artifacts or normal anatomical structures (Table 4).

**Table 3.** The frequency of TP, TN, FP, and FN rates in relation to sensitivity, specificity, PPV, and NPV for readers and AI.

	TP	TN	FP	FN	Sensitivity % (95% CI)	Specificity % (95% CI)	PPV (%) (95% CI)	NPV (%) (95% CI)
Reader 1	152	3705	21	24	86.4 (80.4–91.1)	99.4 (99.1–99.7)	87.9 (82.0–92.3)	99.4 (99.0–99.6)
Reader 2	168	3709	17	8	95.5 (91.2–98.0)	99.5 (99.3–99.7)	90.8 (85.7–94.6)	99.8 (99.6–99.9)
AI A	108	3621	105	68	61.4 (53.7–68.6)	97.2 (96.6–97.7)	50.7 (43.8–57.6)	98.2 (97.7–98.6)
AI B	112	3632	94	64	63.6 (56.1–70.7)	97.5 (96.9–98.0)	54.4 (47.3–61.3)	98.3 (97.8–98.7)
AI C	159	3687	39	17	90.3 (85.0–94.3)	99.0 (98.6–99.3)	80.3 (74.1–85.6)	99.5 (99.3–99.7)
Reader 1 + AI A	158	3705	21	18	89.8 (84.3–93.8)	99.4 (99.1–99.7)	88.3 (82.6–92.6)	99.5 (99.2–99.7)
Reader 2 + AI A	171	3709	17	5	97.2 (93.5–99.1)	99.5 (99.3–99.7)	91.0 (85.9–94.6)	99.9 (99.7–100.0)
Reader 1 + AI B	155	3705	21	21	88.1 (82.3–92.5)	99.4 (99.1–99.7)	88.1 (82.3–92.5)	99.4 (99.1–99.7)
Reader 2 + AI B	171	3709	17	5	97.2 (93.5–99.1)	99.5 (99.3–99.7)	91.0 (85.9–94.6)	99.9 (99.7–100.0)
Reader 1 + AI C	169	3705	21	7	96.0 (92.0–98.4)	99.4 (99.1–99.7)	88.9 (83.6–93.0)	99.8 (99.6–99.9)
Reader 2 + AI C	174	3709	17	2	98.9 (96.0–99.9)	99.5 (99.3–99.7)	91.1 (86.1–94.7)	99.9 (99.8–100.0)

*Note.* The human-AI combination was evaluated using an idealized model, where a case was classified as positive if an ICH was identified by the human reader (TPs and FPs) or (logical OR) correctly identified by the AI (only TPs). All other cases were classified as negative, including FP cases identified by AI.

AI: artificial intelligence; CI: confidence interval; FN: false negative; FP: false positive; NPV: negative predictive value; PPV: positive predictive value; TN: true negative; TP: true positive.

**Table 4.** Factors contributing to FPs: AI detection of ICH on non-contrast head CT.

	All structures^a^	Falx/sinus	Meningioma^b^	Parenchyma	Surgical material/thickened dura	Artifact	Vessels	Calcifications	Unidentified^c^	Other
Total	247	99	31	29	12	17	5	13	17	24
AI-A	110 (44.5)	85 (85.9)	7 (22.6)	4 (13.8)	2 (16.7)	6 (35.3)	2 (40.0)	1 (7.7)	0 (0.0)	3 (12.5)
AI-B	98 (39.7)	7 (7.1)	10 (32.3)	23 (79.3	6 (50.0)	11 (64.7)	2 (40.0)	6 (46.2)	17 (100.0)	16 (66.7)
AI-C	39 (15.8)	7 (7.1)	14 (45.2)	2 (6.9)	4 (33.3)	0 (0.0)	1 (20.0)	6 (46.2)	0 (0.0)	5 (20.8)

Values are given as n (%).

a The total number of FP structures can exceed the number of FP examinations reported in Table 3 because a single examination can contain multiple distinct anatomical artifacts or FP regional flags.

b Meningiomas were not included in the final study cohort.

c The “Unidentified” category represents true FP bounding boxes generated by the AI algorithm where our secondary expert consensus review could find no clear correlating anatomical structure, calcification, or apparent imaging artifact to explain the trigger.

AI: artificial intelligence; FP: false positive; ICH: intracranial hemorrhage.

AI-C's performance was in strong agreement with the adjudicated reference standard, indicated by κ 0.84 (95% confidence interval [CI] = 0.80–0.88). In contrast, AI-A and B showed only moderate agreement with the adjudicated reference standard: κ = 0.53 (95% CI = 0.47–0.59) and κ = 0.57 (95% CI = 0.50–0.63), respectively. Compared to reader 1, AI-A and AI-B showed moderate agreement (κ = 0.52 (95% CI = 0.46–0.58) and κ = 0.59 (95% CI = 0.53–0.65) while AI-C again displayed an almost perfect agreement with κ 0.81 (95% CI = 0.77–0.85).

Mathematically pooling reader 1 with AI-C increased sensitivity to 96.0% while maintaining specificity (99.4%; *P* < 0.001) (Tables 3 and 5). This AI-C + reader 1 combination demonstrated statistically significant superiority in sensitivity compared to standalone reader 1, AI-A, and AI-B (Table 5). However, the combination failed to show superiority over reader 2 alone, i.e. the results after double reading. Combining reader 2 with AI-C increased sensitivity to 98.9% while maintaining specificity (99.5%) (Table 3). This AI-C + reader 2 combination showed statistically significant superiority over all standalone readers and algorithms (Table 5).

**Table 5.** Superiority test *P* values for pairwise reader sensitivity comparisons in assessment of ICH.

	Reader 1	Reader 2	AI-A	AI-B	AI-C	Reader 1 + AI-A	Reader 2 + AI-A	Reader 1 + AI-B	Reader 2 + AI-B	Reader 1 + AI-C	Reader 2 + AI-C
Reader 1		1	**<0**.**001**	**<0**.**001**	0.94	1	1	1	1	1	1
Reader 2	**<0**.**001**		**<0**.**001**	**<0**.**001**	**0**.**039**	**0**.**011**	1	**0**.**002**	1	0.73	1
AI-A	1	1		0.88	1	1	1	1	1	1	1
AI-B	1	1	0.24		1	1	1	1	1	1	1
AI-C	0.12	0.99	**<0**.**001**	**<0**.**001**		0.5	1	0.28	1	1	1
Reader 1 + AI-A	**0**.**016**	1	**<0**.**001**	**<0**.**001**	0.66		1	0.19	1	1	1
Reader 2 + AI-A	**<0**.**001**	0.12	**<0**.**001**	**<0**.**001**	**0**.**006**	**<0**.**001**		**<0**.**001**	0.5	0.38	0.97
Reader 1 + AI-B	0.12	1	**<0**.**001**	**<0**.**001**	0.84	0.97	1		1	1	1
Reader 2 + AI-B	**<0**.**001**	0.12	**<0**.**001**	**<0**.**001**	**0**.**006**	**<0**.**001**	0.5	**<0**.**001**		0.38	0.97
Reader 1 + AI-C	**<0**.**001**	0.50	**<0**.**001**	**<0**.**001**	**0**.**001**	**0**.**002**	0.83	**<0**.**001**	0.83		1
Reader 2 + AI-C	**<0**.**001**	**0.016**	**<0**.**001**	**<0**.**001**	**<0**.**001**	**<0**.**001**	0.19	**<0**.**001**	0.19	**0.031**	

Values are given as *P* values derived from one-sided exact binomial tests for superiority, comparing the performance of the reader listed in the row against the reader in the column. *P* < 0.05 (in bold) indicates that the Row Reader is statistically superior to the Column Reader.

Al: artificial intelligence; ICH: intracranial hemorrhage; readers 1 and 2: individual human readers.

### Superiority analysis comparing pairwise reader sensitivity for ICH detection

Reader 2 demonstrated significantly higher sensitivity than reader 1 (*P* < 0.001). Both human readers were statistically significantly superior to AI-A and AI-B (all *P* < 0.001). Reader 2 indicated statistically significant superiority to AI-C (*P* = 0.039), whereas no statistically significant difference was observed between reader 1 and AI-C (reader 1 superiority *P* = 0.94; AI-C superiority *P* = 0.12). None of the standalone AI systems showed statistically significant superiority over either human reader. AI-A and AI-B did not differ statistically significantly from each other, whereas AI-C differed statistically significantly from both AI-A and AI-B (*P* < 0.001).

The combination of reader 1 + AI-A, reader 1 + AI-B, and reader 1 + AI-C showed varying results, with statistically significant superiority to reader 1 demonstrated for reader 1 + AI-A (*P* = 0.016) and reader 1 + AI-C (*P* < 0.001). The combination reader 2 + AI-C was statistically significantly superior to reader 2 alone (*P* = 0.016), whereas reader 2 + AI-A and reader 2 + AI-B (*P* = 0.12 and *P* = 0.12, respectively) did not show statistically significant superiority. Notably, all hybrid combinations except reader 1 + AI-B showed statistically significant superiority to reader 1 alone. All hybrid human-AI combinations, except for reader 1 + AI-A and reader 1 + AI-B, showed statistically significant superiority to the standalone AI systems, suggesting that integrating AI with human interpretation can substantially improve sensitivity compared with AI alone.

## Discussion

The aim of the present study was to evaluate and compare the diagnostic accuracy of three AI algorithms for ICH detection. The combined human-AI performance maps an idealized upper bound of potential baseline performance rather than a live clinical workflow. This model assumes a human-in-the-loop triage configuration where the physician flawlessly accepts all added true-positive indications discovered by the AI, yet perfectly rejects 100% of the false alarms generated by the software. Consequently, although sensitivity improves across combined models, the system-level specificity remains pinned to the human reader's baseline. This design isolates the maximum potential detection yield without penalizing specificity, but it does not simulate downstream real-world human-AI interaction factors such as alert fatigue, workflow friction, or automation bias.

We observed significant differences in performance metrics between the three algorithms. Only one algorithm (AI-C) demonstrated performance on par with reader 1 in identifying ICH. Still, even though AI-A and AI-B demonstrated lower sensitivity than AI-C when evaluated independently, they may retain therapeutic utility in a complementary role. Because different architectures rely on varying edge-detection matrices and internal voxel features, they often produce non-overlapping error fields. This enables a synergistic effect where joint oversight facilitates complementary detection. When analyzing unique patient exams, AI-C caught an additional 17 human-missed intracranial hemorrhages (152 to 169 TPs), yielding a high NPV (99.8%) that reinforces clinical safety margins in a first-line diagnostic setting. Increased performance for both AI and human readers through complementary collaboration has been reported by other researchers across a range of medical specialties.^15–17^

It was observed that when the AI algorithms misclassified exams as positive (false positives), it often did so in a predictable manner, meaning that in certain contexts false positives are not entirely random but correlate with specific clinical and imaging features. For instance, with AI-A, the most frequent error involved misclassifying the falx cerebri as a suspected hemorrhage. The observation of AI's tendency to misclassifications due to particular error drivers is consistent with observations made by other researchers.^18–21^

The performance of AI-C matched that of the leading algorithms in a recent review.^1,22^ Given the potentially serious consequences of ICH, sensitivity and NPV are the most important outcome measures in a clinical context. The negative aspects of false positives concerning ICH are relatively easy to mitigate with a follow-up examination or an extended observation period. It is therefore interesting to note that AI-C had both higher sensitivity and essentially unchanged NPV compared to reader 1. False-positive results represent a clinical concern in older adults, e.g. as the decision to withhold anticoagulants can result in severe adverse events. Although AI-C demonstrated a lower PPV due to nearly double the false-positive rate of human readers, many of these errors were caused by obvious artifacts or normal anatomy that a radiologist would easily dismiss.

AI-C was the most effective model, catching 17/24 (71%) of reader 1’s misses and 6/8 (75%) of reader 2’s misses. In contrast, AI-A and AI-B performed consistently poorly across both cohorts. AI-A caught only 3/24 (13%) of reader 1’s misses and 2/8 (25%) of reader 2’s misses, while AI-B caught 3/24 (13%) and 3/8 (38%) of the human readers' misses, respectively. Notably, the false negatives overlooked by human readers primarily involved very small shallow subdural hematomas located above the brain convexity, aligning with human errors documented in previous research.^4,23^ The fact that AI and human radiologists often make non-overlapping errors can be advantageous, because it enables so-called complementary detection. Since AI and humans fail in different ways, their collaboration increases the likelihood that errors from one are caught by the other leading to more efficient, accurate, and safe diagnosis.^15,18,24^ This is particularly true in high-volume, first-line settings where the prevalence of conditions like ICH is relatively low, and scans are frequently interpreted by radiologists in training. Leveraging both AI and human oversight appears to enhance diagnostic accuracy.

One potential limitation of this study is that the all-negative cohort did not undergo independent adjudication beyond routine double clinical readings. Although all examinations were interpreted by board-certified radiologists, the possibility remains that subtle ICHs were missed by both clinical readers and the AI. Such shared false negatives could theoretically lead to a marginal overestimation of specificity and NPV. Nevertheless, given our rigorous double-reading process and institutional quality assurance measures, the risk of missing clinically significant ICH is likely minimal. Future research employing an independent expert panel for systematic re-review would further validate these findings.

AI is designed specifically to detect ICH, whereas radiologists must assess the entire CT scan. Our results show that a theoretical integration of AI with a radiologist can improve ICH detection, though the final diagnosis remains dependent on the radiologist's interpretation and experience. Prior studies report high agreement (∼95%) between general radiologists and neuroradiologists in emergency head CT interpretation, with low rates of clinically significant errors.^25^ Gains in accuracy beyond core training appear modest and inconsistent, additional experience tends to improve confidence and management decisions rather than detection of common high-contrast findings such as ICH.^26–29^ Consistent with these high baseline accuracies, adding AI-C increased first-reader sensitivity from 86.4% to 96.0% (*P* < 0.001), mainly by identifying additional true positives.

We assessed whether AI-C combined with a first reader could match or surpass double reading, potentially reducing the need for a second radiologist. Although performance was comparable, the combined radiologist/AI result reflects a theoretical upper bound assuming ideal complementary use and does not account for correlated errors or workflow constraints. The study was underpowered to prove superiority. Notably, double reading is typically retrospective, whereas prospective AI provides real-time support with greater potential to influence acute management.

Our findings also highlight performance variability and the scarcity of studies comparing multiple algorithms on the same dataset. Reliance on manufacturer claims or existing literature, often inadequate,^22^ may yield suboptimal choices in clinical practice. This risk is illustrated by our finding that two of the three tested algorithms underperformed compared to the best-performing model, AI-C. However, performance may vary across institutions depending on data, populations, and workflows. We therefore recommend local validation before clinical implementation.

Despite its retrospective design, the study has strengths. Consecutive cases with minimal exclusions reduced selection bias, and a rigorous adjudicated reference standard was applied. Negative classifications required unanimous agreement among five independent readers (two radiologists and three AI tools), with discrepant cases undergoing structured secondary review by a board-certified radiologist and, when needed, a neuroradiologist. To our knowledge, such a rigorous reference standard has not previously been applied to real-world clinical datasets in this field.

The present study has some limitations. As a single-center study, generalizability to other populations and practices may be limited. The AI algorithms were tested on a small number of scanners from one manufacturer, potentially restricting applicability. However, prior evidence suggests AI performance is robust to variations in imaging quality and technique.^4^ Without an independent gold standard, we used an adjudicated consensus reference standard, introducing possible incorporation bias, and unanimously negative cases were not re-adjudicated. Finally, confidentiality requests from two of the three vendors reduced transparency and may limit external validation and scientific scrutiny. None of the individual AI models surpassed human readers in sensitivity, with reader 2 performing best independently. However, combining AI, particularly AI-C, with human assessment significantly improved sensitivity in several scenarios, outperforming reader 1 alone and even enhancing reader 2's performance. Overall, the results indicate a synergistic effect between human readers and AI, enabling higher sensitivity than either could achieve alone.

In conclusion, we observed significant performance differences among three commercially available algorithms, with only one matching the performance of a single-reader radiologist. These findings emphasize the need for thorough clinical validation before implementation. Combining the algorithm with a first reader improved sensitivity without reducing specificity. As a next step, we will integrate AI-C into our clinical workflow. The real-world interaction between radiologists and AI will be crucial, and some performance decline compared to study conditions is expected. This will be evaluated in a future prospective study.
